# Vagus Nerve Stimulation Inhibits DNA and RNA Methylation in a Rat Model of Pilocarpine‐Induced Temporal Lobe Epilepsy

**DOI:** 10.1111/cns.70484

**Published:** 2025-06-18

**Authors:** Zhonghua Xiong, Jing Zhang, Qinqin Deng, Minghui Wang, Tianfu Li

**Affiliations:** ^1^ Department of Neurosurgery, Sanbo Brain Hospital Capital Medical University Beijing China; ^2^ Laboratory for Clinical Medicine Capital Medical University Beijing China

**Keywords:** 5‐hmC, 5‐mC, epilepsy, METTL14, METTL3, vagus nerve stimulation

## Abstract

**Introduction:**

Vagus nerve stimulation (VNS) represents a clinically approved neuromodulatory intervention for managing refractory epilepsy. VNS exhibits antiepileptogenic effects in animal models; the molecular and cellular mechanisms driving these effects have yet to be fully elucidated. Epigenetic alterations, including DNA and RNA methylation, are implicated in the pathogenesis of epilepsy. This study was designed to examine whether VNS inhibits DNA/RNA methylation in temporal lobe epilepsy.

**Methods:**

A rat model of temporal lobe epilepsy induced by intraperitoneal injection of lithium chloride‐pilocarpine was used. Naïve control group, Pilo groups (8 and 12 weeks after pilocarpine respectively), VNS group (VNS stimulation for 4w and 8w respectively), and Sham group (VNS off control group, with VNS implanted but not turned on for 4w and 8w respectively). The rats were monitored by video‐EEG. DNMT1 (DNA methyltransferase‐1), DNMT3A (methyltransferase‐3A), 5‐hmC (5‐hydroxymethyl‐cytosine), 5‐mC (5‐methyl‐cytosine), METTL3 (RNA methyltransferase like 3), METTL14 (RNA methyltransferase like 14) were quantified via immunohistochemistry and Western blot.

**Results:**

Pilocarpine‐induced epileptic animals were characterized by overexpression of 5‐mC, DNMT1, DNMT3A, METTL3, and METTL14, and a decrease of 5‐hmC. Compared to the Pilo group, VNS significantly inhibited the overexpression of 5‐mC, DNMT1, DNMT3A, METTL3, and METTL14, reduced the downregulation of 5‐hmC, and attenuated spontaneous recurrent seizures (SRSs).

**Conclusions:**

We conclude that VNS reduces the number of SRSs in a rat model of epilepsy, concurrently reducing the expression of 5‐mC, DNMT1, DNMT3A, METTL3, and METTL14, as well as the increase of 5‐hmC. These findings suggest that epigenetic modifications, specifically the suppression of DNA and RNA methylation, could constitute a potential target of VNS‐mediated antiepileptic effects.

## Graphical abstract

Vagus nerve stimulation (VNS) reduces the number of SRSs in rats with epilepsy with concomitant decrease of 5‐mC, DNMT1, DNMT3A, METTL3, and METTL14, as well as increase of 5‐hmC. Inhibition of DNA/RNA methylation might be the potential targets of VNS for the treatment of epilepsy. Created with biorender.com.

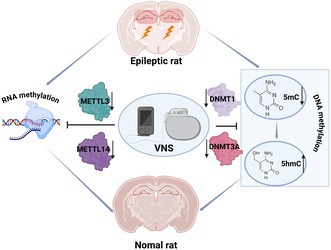

## 1 Introduction

Epilepsy affects over 70 million individuals globally, with approximately one‐third experiencing pharmacoresistant forms [1]. These patients often endure persistent, disabling seizures, poor quality of life, and a spectrum of cognitive and psychiatric comorbidities [2]. Although many patients are ineligible for certain treatment options, surgical resection of the epileptogenic zone remains the last resort in patients with pharmacoresistant epilepsy, and 30%–40% of patients with surgery still fail to relieve their seizures [3]. As a recognized palliative approach, vagus nerve stimulation (VNS) is applied in pharmacoresistant epilepsy cases where resective surgery is not feasible. It results in a more than 50% reduction in seizure frequency in 50%–60% of patients, with 6%–11% attaining complete seizure control [4]. The efficacy of VNS is improved with the extension of treatment time [5, 6, 7, 8]. Of importance, experimental studies indicate that VNS has antiepileptogenic properties [9, 10, 11].

Epigenetic modifications, particularly alterations in DNA methylation, contribute to epileptogenesis by inducing long‐lasting modifications in neuronal excitability. Extensive studies demonstrated that chronic epileptic conditions are associated with increased DNA methylation in both experimental animal models [12, 13] and resected human epileptic tissues [14, 15, 16]. To reversal of these epigenetic changes may halt disease progression [17, 18], therefore, DNA hypermethylation is highlighted as a therapeutic biomarker for epilepsy development [19]. N6‐methyladenosine (m6A) is the most prevalent epigenetic modification involved in post‐transcriptional regulation of mRNA. The m6A methyltransferase METTL3/14 (RNA methyltransferase like 3/14), known as the m6A “writer”, is implicated in epilepsy [20, 21]. Although previous studies have linked DNA methylation to epileptogenesis, the role of RNA methylation (m6A) in epilepsy remains largely unexplored. Moreover, it is still unclear whether VNS exerts its antiepileptic effects through modulation of DNA and RNA methylation.

In the present study, a rat temporal lobe epilepsy model was established and VNS therapy was initiated 4 weeks after status epilepticus induced by pilocarpine to evaluate whether VNS can inhibit seizures through the DNA/RNA methylation‐mediated mechanism. This dual‐level epigenetic analysis provides novel insight into the molecular mechanisms underlying VNS therapy in epilepsy.

## 2 Method and Material

### 2.1 Pilocarpine‐Induced Model of Temporal Lobe Epilepsy

**FIGURE 1:**
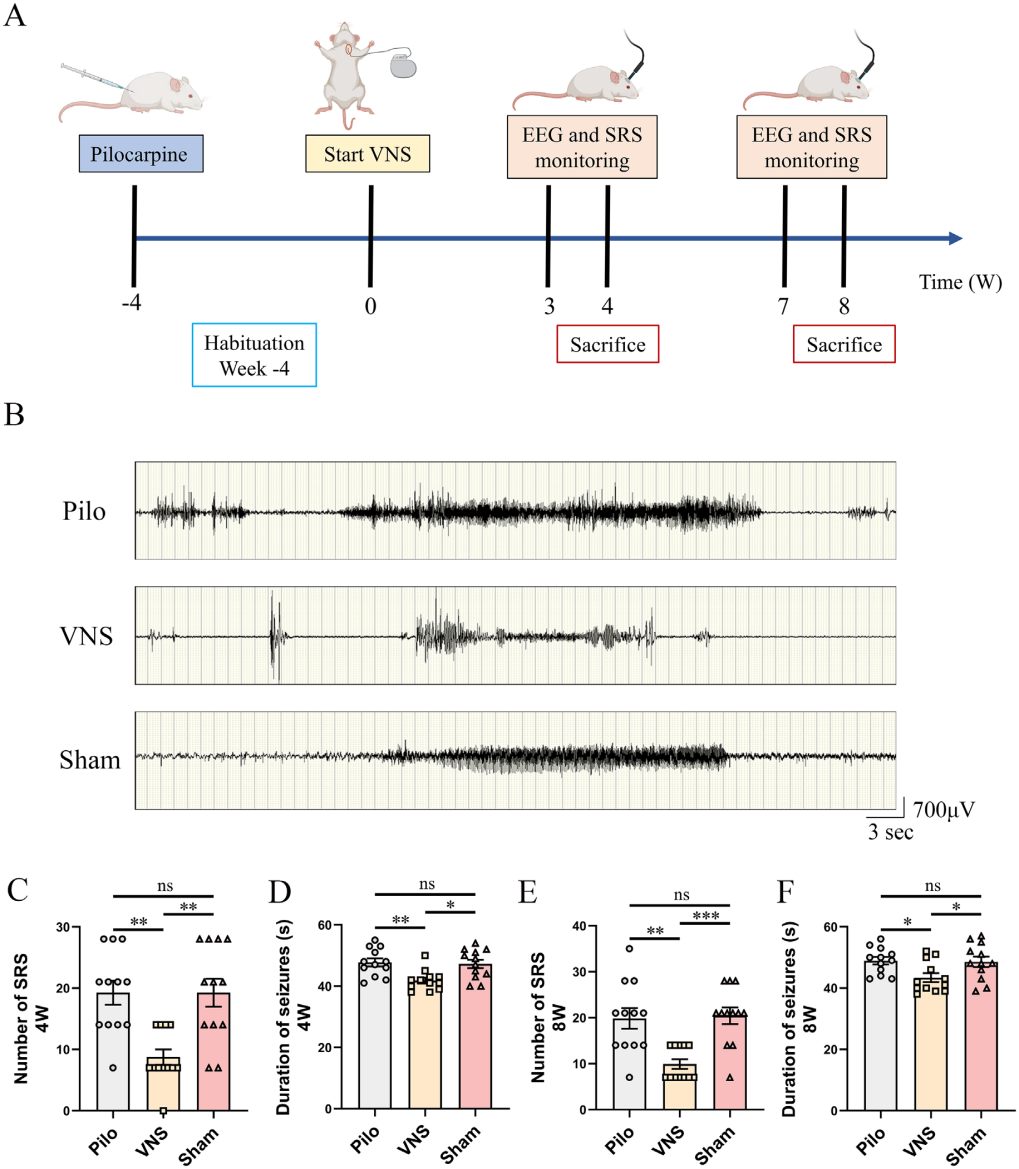
The experimental schedule and results of EEGs. (A) The experimental design and schedule. VNS = Vagus nerve stimulation, SRSs = spontaneous recurrent seizures, EEG = electroencephalogram. (B) Representative EEG recordings from the hippocampus showed spontaneous electrographic seizures in the Pilo, VNS, and Sham in the 4th week. (C, D) The mean number of SRSs and mean seizure duration of the VNS group was significantly shorter than that of the Sham group in the 4th week (*p < 0.05, **p < 0.01, *n* = 12). No significant difference was observed between the Pilo group and the Sham group. (E, F) The mean number of SRSs and mean seizure duration of the VNS group was significantly shorter than that of the Sham group in the 8th week (*p < 0.05, **p < 0.01, ***p < 0.001, *n* = 12). No significant difference was observed between the Pilo group and the Sham group.

Experiments were conducted on Sprague–Dawley rats weighing 260–300 g, housed under a reversed 12:12 h light–dark cycle with unrestricted access to standard diet and water. All handling, housing, stimulation, and surgical procedures were approved by The Capital Medical University Institutional Animal Care and ethics Committee. The experimental rats were randomly divided into groups as follows: Control group (naive rats), Pilo groups (8 and 12 weeks after pilocarpine respectively), VNS groups (VNS stimulation for 4w and 8w respectively) and Sham group (VNS off control group, with VNS implanted but not turned on for 4w and 8w respectively). The adult rats were received lithium chloride (intraperitoneally (i.p.) 127 mg/kg, Sigma) 24 h before pilocarpine. Pilocarpine hydrochloride (50 mg/kg, Sigma) was administered intraperitoneally 30 min after subcutaneous injection of atropine sulfate (1 mg/kg, Sigma). Atropine sulfate was used to block the peripheral muscarinic receptors. Seizure severity was estimated according to Racine's scale. Only rats exhibiting stage IV or V seizures were included in the study. Control animals received equivalent volumes of saline. Diazepam (10 mg/kg; Tianjin JinYao Pharmaceutical Co., China) was administered intraperitoneally 60 min after the onset of status epilepticus (SE) to terminate seizures. Additional doses of diazepam (5 mg/kg) were given as needed to fully suppress SE. The experimental design and schedule are represented in Figure 1A.

### 2.2 VNS Surgery

In brief, rats were anesthetized with isoflurane. At the cervical level, the left vagus nerve was surgically exposed and separated from adjacent tissues. A silicone spiral cuff electrode with platinum contacts (Ri Shena, VNS 201, China) was positioned around the nerve, and stimulation was initiated seven days after implantation (parameters: the out current was 1.0 mA, the signal on time was 30 s, and the signal off time as 5 min).

### 2.3 EEG Recording

Following induction of anesthesia using a gas mixture comprising 68.5% N_2_O, 30% O_2_, and 1.5% isoflurane, a bipolar stainless‐steel electrode (0.20 mm diameter; Plastics One Inc.) was stereotactically placed into the left hippocampal CA1 subregion (coordinates: AP −4.56 mm, ML +2.7 mm, DV −3.0 mm) and affixed with dental cement. A reference electrode was implanted into the cerebellum. Video‐EEG recordings were performed using the NicoletOne EEG system (Natus) with frequency filters set at 1–70 Hz and a chart speed of 15 mm/s. Recordings were conducted for 8 h daily (from 10:00 a.m. to 6:00 p.m.) across seven consecutive days. At either week 4 or week 8 post‐implantation, spontaneous recurrent seizures (SRSs) graded 3–5 on the Racine scale [22], along with seizure durations, were monitored and documented.

### 2.4 Histopathological Assessment

After induction of deep anesthesia, half of the rats were transcardially perfused with 4% paraformaldehyde. Coronal brain sections encompassing the hippocampal region were prepared and processed for immunohistochemistry. The staining protocol followed standard procedures as described in earlier studies [23, 24, 25]. Primary antibodies employed in this study included: anti‐5‐mC (Mouse monoclonal antibody A‐1014, 1:500, Epigentek, Farmingdale), anti‐5‐hmC (Rabbit polyclonal antibody 39,769, 1:3000, Active Motif, Carlsbad), anti‐METTL3 (Rabbit A8370, 1:100, ABclonal, Woburn) and anti‐METTL14 (Rabbit A8530, 1:100, ABclonal, Woburn). Sections were incubated overnight at 4 °C with a cocktail of primary antibodies, followed by a 1‐h room temperature incubation with the corresponding HRP‐conjugated secondary antibodies. Immunoreactivity was visualized using the Polink‐1 HRP DAB Detection Kit (ZSGB‐BIO, Beijing, China), and DAB was applied for color development. Hematoxylin counterstaining was performed thereafter. Imaging was completed using a Leica DM3000 LED microscope (Leica Microsystems, Germany). Semi‐quantitative evaluation was conducted according to modified protocols from prior studies [26, 27]. Signal intensities were measured within a defined 2 × 3 mm^2^ region using Kodak imaging software. Levels of 5‐mC, 5‐hmC, METTL3, and METTL14 were quantified in arbitrary units and expressed as percentage changes relative to control values.

### 2.5 Western Blotting

Western blotting was conducted based on previously reported protocols [28]. Hippocampal tissues were homogenized in lysis buffer, and protein samples (30 μg per lane) were separated via SDS‐PAGE. Proteins were then transferred onto 0.22 μm PVDF membranes (Millipore, USA) and blocked with 5% skim milk. Membranes were incubated overnight at 4 °C with the following primary antibodies: anti‐DNMT1(Rabbit D63A6, 1:1000, Cell signaling, Beverly), anti‐DNMT3A(Rabbit 3598S, 1:1000, Cell signaling, Beverly), anti‐METTL3 (Rabbit A8370, 1:1000, ABclonal, Woburn), and anti‐METTL14 (Rabbit A8530, 1:500, ABclonal, Woburn). β‐actin (Mouse monoclonal, 1:1000, Cell Signaling Technology) served as a loading control. After incubation with HRP‐conjugated secondary antibodies (1:5000; Applygen Technologies), protein bands were visualized using enhanced chemiluminescence (ECL) reagents. Detection was performed with a LAS‐3000 imaging system (Fujifilm), and band intensities were quantified using ImageJ software.

### 2.6 Statistics Analyses

For all analyses, statistical significance was performed by GraphPad Prism 8.4.2 software (GraphPad Software, San Diego) and SPSS version 27.0, and data were displayed as mean ± SEM. Normality was assessed using the D'Agostino and Pearson omnibus test. Continuous variables with normal distributions were analyzed using an independent t‐test, while those without were evaluated using the Mann–Whitney U‐test. For comparisons among multiple groups, one‐way ANOVA was applied, followed by Bonferroni correction for multiple testing. Statistical significance was defined as *p* < 0.05. All experiments were independently repeated at least three times unless otherwise stated.

## 3 Results

### 3.1 VNS Reduces the Frequency of SRS in Epileptic Rats

To assess the impact of VNS on seizure activity, behavioral episodes and video‐EEG recordings were evaluated at weeks 4 and 8 following stimulation. 12 rats were included in each group. No seizures or interictal discharges were observed in the control group throughout the study. At week 4, the VNS‐treated rats exhibited a significantly lower seizure frequency compared to the Sham group (Figure 1C; 8.75 ± 1.26 vs. 19.25 ± 2.30 episodes/day, *p* < 0.01), while no significant difference was detected between the Pilo and Sham groups. Similarly, seizure duration in the VNS group was markedly reduced relative to the Sham group (Figure 1D; 41.83 ± 0.96 vs. 47.25 ± 1.39 s, *p* < 0.05), with no difference observed between Pilo and Sham animals. At week 8, a comparable pattern was observed. Seizure frequency in the VNS group remained significantly lower than in the Sham group (Figure 1E; 9.92 ± 1.04 vs. 20.42 ± 1.82 episodes/day, *p* < 0.001). The average seizure duration was also significantly shorter in VNS‐treated rats (Figure 1F; 43.42 ± 1.46 vs. 48.58 ± 1.72 s, *p* < 0.05). Again, no significant differences were noted between the Pilo and Sham groups. Collectively, these findings indicate that VNS effectively reduces both the frequency and duration of SRS in this rat model of epilepsy.

### 3.2 VNS Reverses DNA Methylation

#### 3.2.1 VNS Inhibits the Overexpression of 5mC

**FIGURE 2:**
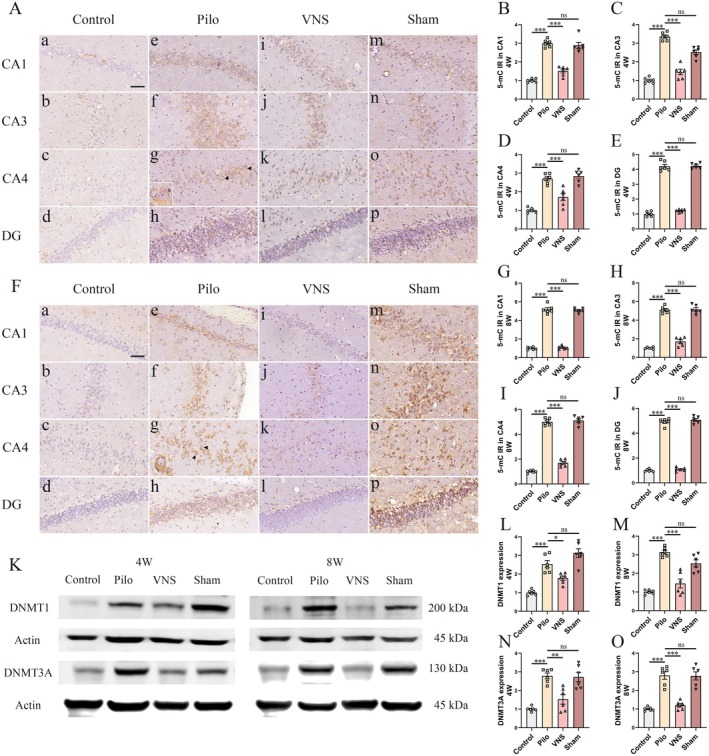
Effect of VNS on 5mC, DNMT1, and DNMT3A expression in the hippocampus. Immunohistochemical staining and western blotting of 5mC were evaluated in the hippocampus at 4th and 8th weeks after SE. (A) Immunoreactivity of 5mC was present in neuronal cells with only weak staining (Aa‐d) in the control group. Compared to the control group, the Pilo group (Ae‐h, inset arrow) showed higher expression of 5mC in the CA1 (B), CA3 (C), CA4 (D) and DG (E) regions (***p < 0.001 respectively). No statistical difference within hippocampal regions was discovered across Sham (Am‐p) and the Pilo groups (*p* > 0.05 respectively). Compared to the Pilo group, significantly lower expression of 5mC in the VNS group (Ai‐l) was detected within hippocampal regions (***p < 0.001 respectively). (F) In the 8 weeks VNS stimulation study, compared to the control group (Aa‐d), the Pilo group (Fe‐h, inset arrow) showed higher expression of 5mC in the CA1 (G), CA3 (H), CA4 (I) and DG (J) regions (***p < 0.001 respectively). No statistical difference within hippocampal regions was discovered across Sham groups (F, m–p) and the Pilo (*p* > 0.05 respectively). Compared to the Pilo group, significantly lower expression of 5mC in the VNS group (Fi‐l) was detected within hippocampal regions (***p < 0.001 respectively). (K) Western blot analysis was performed to quantify the amount of DNMT1 and DNMT3A in total homogenates of the hippocampus from rats in each group. (L–O) Compared with the control group, DNMT1 and DNMT3A levels in the hippocampus were elevated in the Pilo and Sham groups (***p < 0.001). The levels of DNMT1 and DNMT3A in the VNS group were considerably lower than those in the Pilo group (*p < 0.05, **p < 0.01, ***p < 0.001). No significant difference in DNMT1 and DNMT3A levels was detected between the Pilo and Sham groups. All Figures shown were representative examples based on the analysis of rats (*n* = 6) per group. Scale bars = 20 μm (CA1, CA3, CA4 and DG).

In the 4 weeks VNS stimulation study, similar to the previous study [13], immunoreactivity of 5mC was present in sparse neuronal cells with only weak staining (Figure 2A,a–d) in the control group. Compared to the control group, the Pilo group (Figure 2A,e–h, inset arrow) showed higher expression of 5mC in the CA1, CA3, CA4, and DG regions (Figure 2B–E, *p* < 0.001 respectively). No statistical difference within hippocampal regions was discovered across Sham (Figure 2Am–p) and the Pilo groups (Figure 2B–E, *p* > 0.05 respectively). Compared to the Pilo group, significantly lower expression of 5mC in the VNS group (Figure 2A,i–l) was detected within hippocampal regions (Figure 4B–E, *p* < 0.001 respectively). In the 8 weeks VNS stimulation study, compared to the control group, the Pilo group (Figure 2F,e–h, inset arrow) showed higher expression of 5mC in the CA1, CA3, CA4, and DG regions (Figure 2G–J, *p* < 0.001 respectively). No statistical difference within hippocampal regions was discovered across Sham groups (Figure 2F,m–p) and the Pilo (Figure 2G–J, *p* > 0.05 respectively). Compared to the Pilo group, significantly lower expression of 5mC in the VNS group (Figure 2F,i–l) was detected within hippocampal regions (Figure 2G–J, *p* < 0.001 respectively). The increased hippocampal 5mC density observed in epileptic rats was markedly reduced following VNS treatment, as confirmed by quantitative analysis.

#### 3.2.2 VNS Inhibits the Overexpression of DNMT1 and DNMT3A

Western blotting was conducted to assess DNMT1 and DNMT3A protein levels in hippocampal homogenates across all groups (Figure 2K). At week 4, DNMT1 and DNMT3A expression was significantly elevated in the Pilo group compared to controls (Figure 2L, *n* = 6; *p* < 0.001). No significant difference was observed between the Pilo and Sham groups (*p* > 0.05). However, VNS treatment markedly reduced DNMT1 and DNMT3A levels relative to the Pilo group (*p* < 0.05 and *p* < 0.001, respectively). A similar trend was noted at week 8. DNMT1 and DNMT3A expression remained significantly higher in the Pilo group versus controls (Figure 2M, *n* = 6; *p* < 0.001), with no significant differences between the Pilo and Sham groups (*p* > 0.05). Notably, VNS again led to a significant reduction in both proteins when compared to the Pilo group (*p* < 0.05 and *p* < 0.001, respectively). These results suggest that VNS therapy can inhibit the upregulation of DNMT1 and DNMT3A in epileptic rats.

#### 3.2.3 VNS Attenuates the Reduction of 5hmC

**FIGURE 3:**
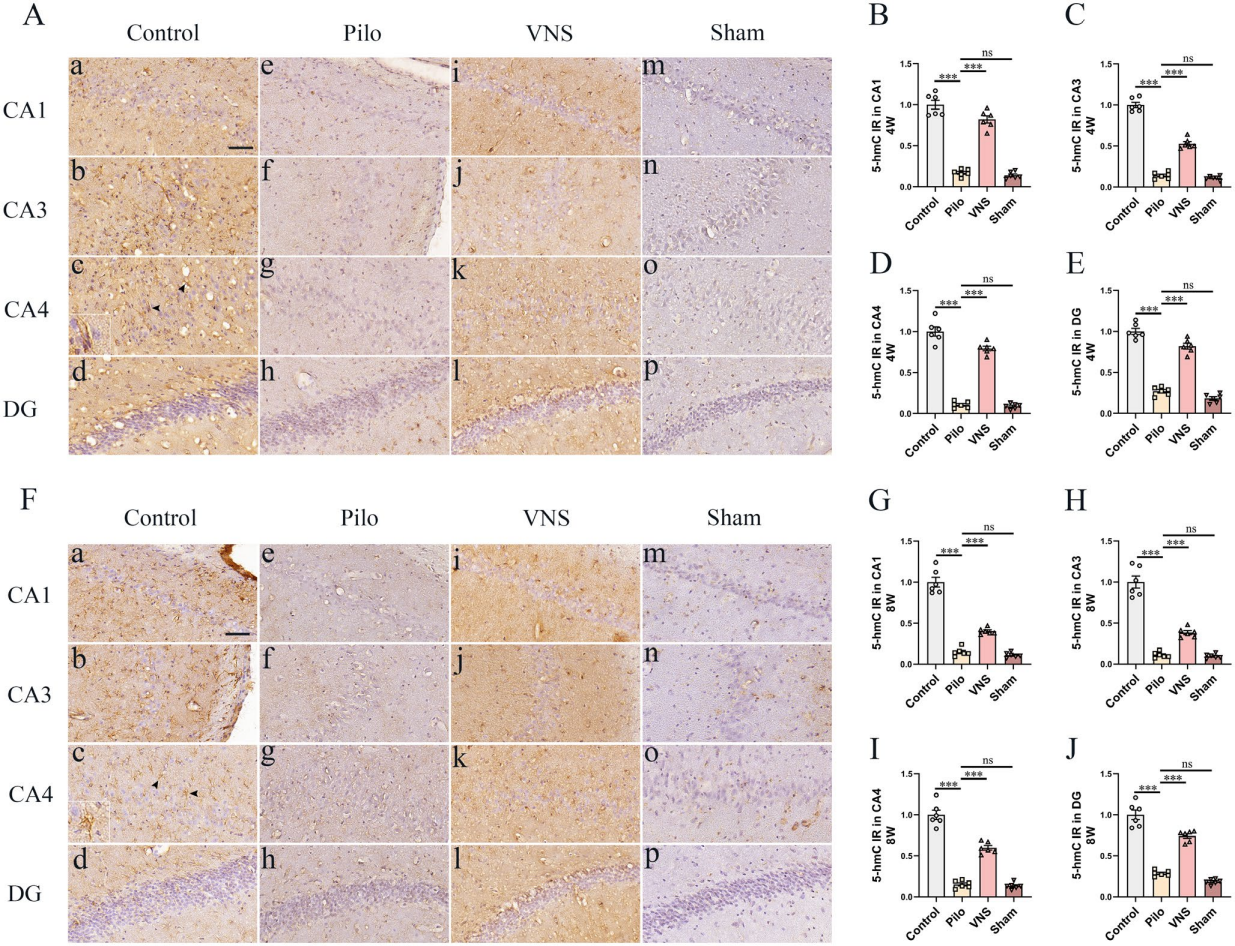
Effect of VNS on 5hmC expression in the hippocampus. Immunohistochemical staining and western blotting of 5hmC were evaluated in the hippocampus at 4th and 8th weeks after SE. (A) Immunoreactivity of 5hmC was present in astrocyte cells. Compared to the control group (A, a–d, inset arrow), the Pilo group (A, e–h) showed lower expression of 5hmC in the CA1 (B), CA3 (C), CA4 (D) and DG (E) regions (***p < 0.001 respectively). No statistical difference within hippocampal regions was discovered across Sham (A, m–p) and the Pilo groups (*p* > 0.05 respectively). Compared to Pilo group, significantly higher expression of 5hmC in the VNS group (A, i–l) was detected within hippocampal regions (***p < 0.001 respectively). (F) In the 8 weeks VNS stimulation study, compared to the control group (F, a–d, inset arrow), the Pilo group (F, e–h) showed lower expression of 5hmC in the CA1 (G), CA3 (H), CA4 (I) and DG (J) regions (***p < 0.001 respectively). No statistical difference within hippocampal regions was discovered across Sham groups (F, m–p) and the Pilo (*p* > 0.05 respectively). Compared to Pilo group, significantly higher expression of 5hmC in the VNS group (F, i–l) was detected within hippocampal regions (***p < 0.001 respectively). All Figures shown were representative examples based on the analysis of rats (*n* = 6) per group. Scale bars = 20 μm (CA1, CA3, CA4 and DG).

Immunohistochemical analysis was performed to evaluate 5hmC levels in brain sections of rats across all groups. At week 4 following VNS stimulation, 5hmC immunoreactivity was predominantly observed in astrocytes within the hippocampus of control rats (Figure 3A,a–d, inset arrow). In contrast, the Pilo group exhibited a marked reduction of 5hmC signals in the CA1, CA3, CA4, and DG regions (Figure 3A,e–h), as confirmed by quantitative analysis (Figure 3B–E; *p* < 0.001 respectively). No significant differences were noted between the Pilo and Sham groups (*p* > 0.05). Importantly, VNS treatment substantially restored 5hmC levels in these hippocampal subfields relative to the Pilo group (Figure 3A,i–l,B–E; *p* < 0.001). Similar results were obtained at week 8. In the control group, robust 5hmC expression remained detectable in hippocampal astrocytes (Figure 3F,a–d, inset arrow), whereas the Pilo group showed significantly decreased 5hmC levels across the same subregions (Figure 3F,e–h; *p* < 0.001). Again, no statistical difference was found between the Pilo and Sham groups (Figure 3G–J; *p* > 0.05). VNS stimulation significantly increased 5hmC expression compared to the Pilo group (Figure 3G–J; *p* < 0.001). These results confirmed that VNS therapy can significantly attenuate the downregulation of 5hmC in epileptic rats.

#### 3.2.4 VNS Reverses m6A Methyltransferase METTL3

**FIGURE 4:**
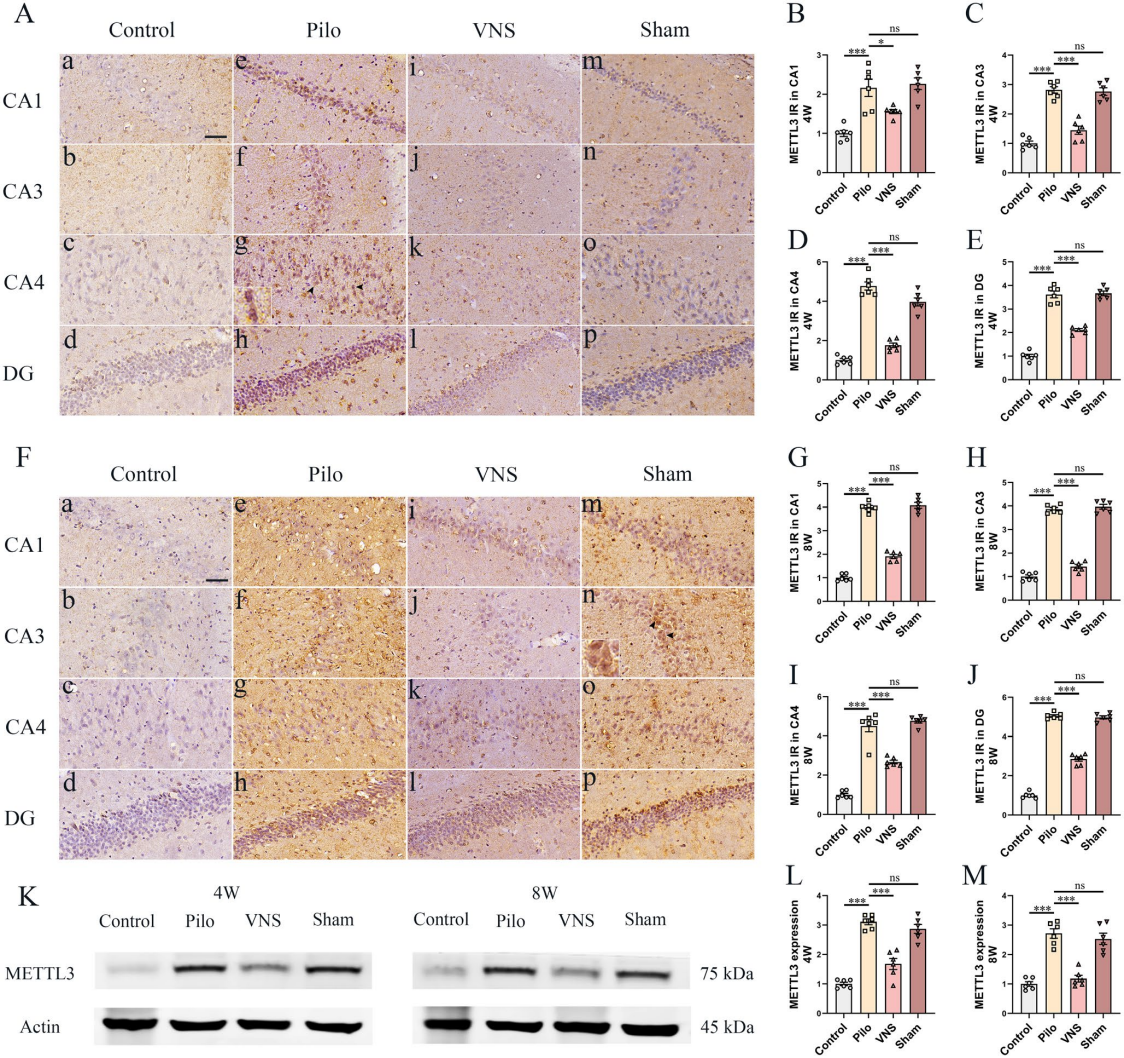
Effect of VNS on the METTL3 expression in hippocampus. Immunohistochemical staining and western blotting of METTL3 were evaluated in the hippocampus at 4th and 8th weeks after SE. (A) Immunoreactivity of METTL3 was present in sparse neuronal cells with only weak staining (A, a–d) in the control group. Compared to the control group, the Pilo group (A, e–h, inset arrow) showed higher expression of METTL3 in the CA1 (B), CA3 (C), CA4 (D) and DG (E) regions (***p < 0.001 respectively). No statistical difference within hippocampal regions was discovered across Sham (A, m–p) and the Pilo groups (*p* > 0.05 respectively). Compared to the Pilo group, significantly lower expression of METTL3 in the VNS group (A, i–l) was detected within hippocampal regions (*p < 0.05, ***p < 0.001 respectively). (F) In the 8 weeks VNS stimulation study, compared to the control group, the Pilo group (F, e–h) showed higher expression of METTL3 in the CA1 (G), CA3 (H), CA4 (I) and DG (J) regions (***p < 0.001 respectively). No statistical difference within hippocampal regions was discovered across Sham groups (F, m–p, inset arrow) and the Pilo (*p* > 0.05 respectively). Compared to the Pilo group, significantly lower expression of METTL3 in the VNS group (F, i–l) was detected within hippocampal regions (***p < 0.001 respectively). (K) Western blot analysis was performed to quantify the amount of METTL3 in total homogenates of the hippocampus from rats in each group. (L–M) Compared with the control group, METTL3 levels in the hippocampus were elevated in the Pilo and Sham groups (***p < 0.001). The levels of METTL3 in the VNS group were considerably lower than those in the Pilo group (***p < 0.001). No significant difference in METTL3 levels was detected between Pilo and Sham groups. All Figures shown were representative examples based on the analysis of rats (*n* = 6) per group. Scale bars = 20 μm (CA1, CA3, CA4 and DG).

At week 4 following VNS stimulation, immunoreactivity of METTL3 was present in sparse neuronal cells with weak staining in control group (Figure 4A,a–d). Compared to the control group, the Pilo group (Figure 4A,e–h, inset arrow) showed higher expression of METTL3 in the CA1, CA3, CA4 and DG regions (Figure 4B–E, *p* < 0.001 respectively). No significant difference within hippocampal regions was discovered across Sham (Figure 4A,m–p) and the Pilo groups (Figure 4B–E, *p* > 0.05 respectively). The levels of METTL3 in the VNS group (Figure 4A,i–l) were considerably lower than that in the Pilo group (Figure 4B–E, *p* < 0.05, *p* < 0.001, *p* < 0.001, *p* < 0.001 respectively). Western blotting was used to quantify METTL3 expression in hippocampal homogenates across all groups (Figure 4K). METTL3 levels were significantly elevated in the Pilo group compared to controls (Figure 4L, p < 0.001), while no significant difference was observed between the Pilo and Sham groups (*p* > 0.05). Notably, VNS treatment resulted in a marked reduction in METTL3 expression relative to the Sham group (*p* < 0.001, Figure 4L).

At week 8 following VNS stimulation, compared to the control group (Figure 4F,a–d), the Pilo group (Figure 4F,e–h) showed higher expression of METTL3 in the CA1, CA3, CA4 and DG regions (Figure 4G–J, *p* < 0.001 respectively). No statistical difference within hippocampal regions was discovered across Sham (Figure 4F,m–p) and the Pilo groups (Figure 4G–J, *p* > 0.05 respectively). The immunoreactivity of METTL3 in the hippocampus was markedly reduced in the VNS group compared to the Pilo group (Figure 4F,i–l, *p* < 0.001 respectively). Consistently, Western blot analysis confirmed these findings (Figure 4K). METTL3 protein levels were significantly elevated in the Pilo group relative to controls (Figure 4M; *p* < 0.001), while no statistical difference was observed between the Pilo and Sham groups (*p* > 0.05). Notably, VNS treatment resulted in a significant decrease in METTL3 expression when compared to the Sham group (Figure 4M; *p* < 0.001). These results demonstrated that VNS therapy can significantly inhibit the upregulation of METTL3 in epileptic rats.

#### 3.2.5 VNS Reverses m6A Methyltransferase METTL14

**FIGURE 5:**
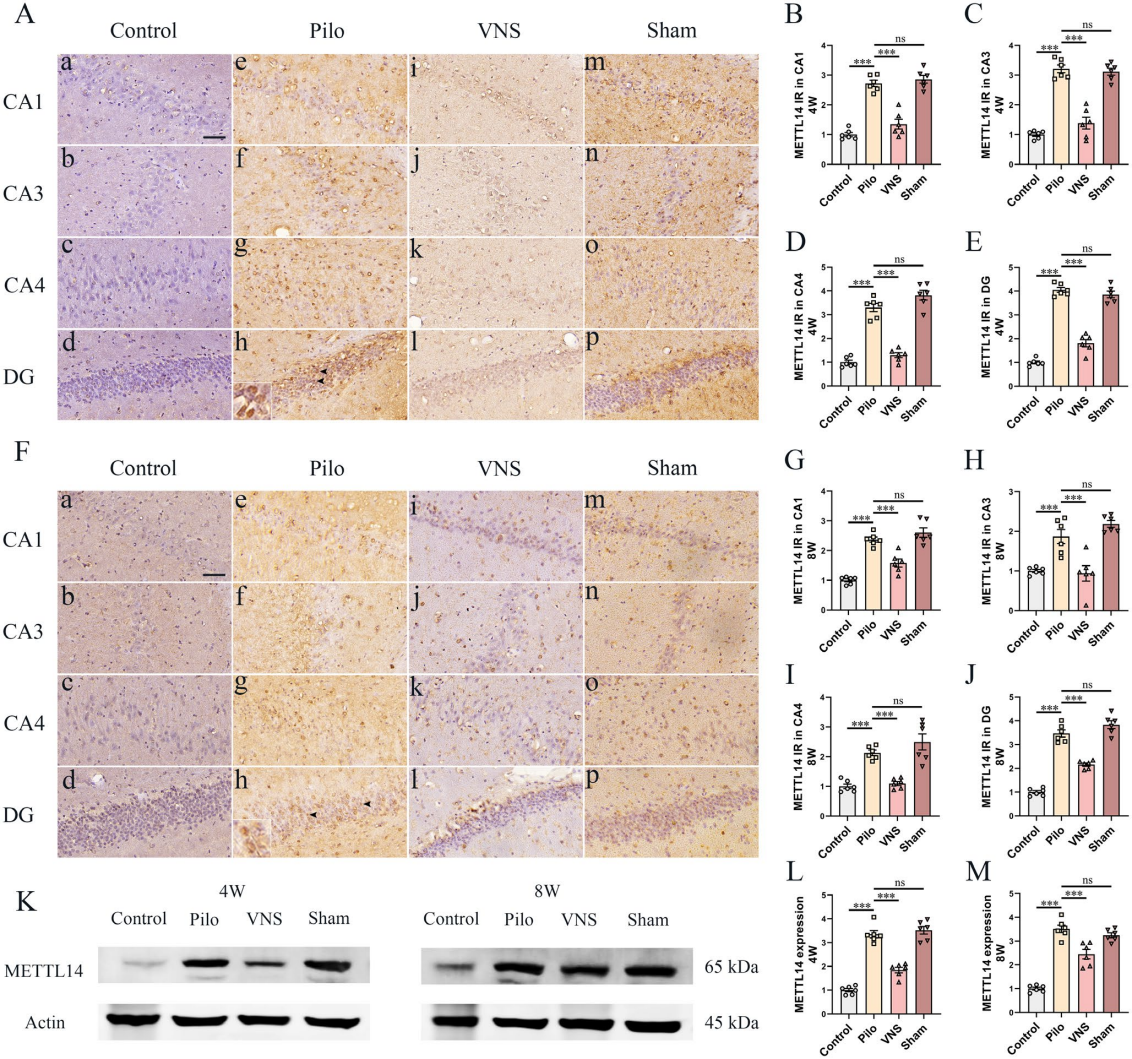
Effect of VNS on the METTL14 expression in thehippocampus. Immunohistochemical staining and western blotting of METTL14 were evaluated in the hippocampus at 4th and 8th weeks after SE. (A) Immunoreactivity of METTL14 was present in sparse neuronal cells with only weak staining (A, a–d) in control group. Compared to the control group, the Pilo group (A, e–h, inset arrow) showed higher expression of METTL14 in the CA1 (B), CA3 (C), CA4 (D) and DG (E) regions (***p < 0.001 respectively). No statistical difference within hippocampal regions was discovered across Sham (A, m–p) and the Pilo groups (*p* > 0.05 respectively). Compared to Pilo group, significantly lower expression of METTL14 in the VNS group (A, i–l) was detected within hippocampal regions (***p < 0.001 respectively). (F) In the 8 weeks VNS stimulation study, compared to the control group (F, a–d), the Pilo group (F, e–h, inset arrow) showed higher expression of METTL14 in the CA1 (G), CA3 (H), CA4 (I) and DG (J) regions (***p < 0.001 respectively). No statistical difference within hippocampal regions was discovered across Sham groups (F, m–p) and the Pilo (*p* > 0.05 respectively). Compared to Pilo group, significantly lower expression of METTL14 in the VNS group (F, i–l) was detected within hippocampal regions (***p < 0.001 respectively). (K) Western blot analysis was performed to quantify the amount of METTL14 in total homogenates of the hippocampus from rats in each group. (L–M) Compared with control group, METTL14 levels in the hippocampus were elevated in the Pilo and Sham groups (***p < 0.001). The levels of METTL14 in the VNS group were considerably lower than that in the Pilo group (***p < 0.001). No significant difference in METTL14 levels was detected between Pilo and Sham groups. All Figures shown were representative examples based on the analysis of rats (*n* = 6) per group. Scale bars = 20 μm (CA1, CA3, CA4 and DG).

At week 4 following VNS stimulation, immunoreactivity of METTL14 was present in sparse neuronal cells with weak staining in the control group (Figure 5A,a–d). Compared to the control group, the Pilo group (Figure 5A,e–h, inset arrow) showed higher expression of METTL14 in the CA1, CA3, CA4, and DG regions (Figure 5B–E, *p* < 0.001 respectively). No significant difference within hippocampal regions was discovered across Sham (Figure 5A,m–p) and the Pilo groups (Figure 5B–E, *p* > 0.05 respectively). The METTL14 expression in the hippocampus was significantly reduced in the VNS group compared to the Pilo group (Figure 5A,i–l, *p* < 0.001). This reduction was further supported by Western blot analysis (Figure 5K). METTL14 protein levels were markedly elevated in the Pilo group relative to controls (Figure 5L, *p* < 0.001), with no significant difference observed between the Pilo and Sham groups (*p* > 0.05). Notably, VNS treatment led to a significant decrease in METTL14 levels compared to the Sham group (Figure 5L, *p* < 0.001).

At week 8 following VNS stimulation, compared to the control group (Figure 5F,a–d), the Pilo group (Figure 5F,e–h, inset arrow) showed higher expression of METTL14 in the CA1, CA3, CA4, and DG regions (Figure 5G–J, *p* < 0.001 respectively). No significant difference within hippocampal regions was discovered across Sham (Figure 5F,m–p) and the Pilo groups (Figure 5G–J, *p* > 0.05 respectively). Compared to the Pilo group, METTL14 levels were significantly downregulated in the VNS group (Figure 5F,i–l, *p* < 0.001). These findings were corroborated by Western blot results (Figure 5K), which showed markedly elevated METTL14 levels in the Pilo group relative to controls (Figure 5M, *p* < 0.001). No statistical difference was detected between the Pilo and Sham groups (*p* > 0.05). Notably, VNS treatment led to a significant reduction in METTL14 expression compared to the Sham group (Figure 5M, *p* < 0.001). Collectively, these results indicate that VNS effectively counteracts METTL14 upregulation associated with epilepsy.

## 4 Discussion

### 4.1 VNS Therapy Inhibits Epileptic Seizures

VNS has been widely recognized as a palliative therapeutic option for patients with pharmacoresistant epilepsy who are not suitable candidates for surgical resection. Epileptogenesis refers to the process by which brain tissue acquires the ability to generate spontaneous seizures, leading either to the initial onset of epilepsy or to its progression following diagnosis—a phenomenon often referred to as secondary epileptogenesis [29]. In the early stages of the pilocarpine model, a maturation phase of approximately 8 weeks follows the first detected SRS, during which seizure activity can progressively involve additional brain regions. This accelerating process is referred to as secondary epileptogenesis [30]. Specifically, it has been demonstrated that a “mirror focus” can develop in brain regions contralateral or ipsilateral to the original epileptogenic zone, indicating the spread of epileptogenic potential to previously unaffected areas [31]. Previous experimental study demonstrated that VNS has antiepileptogenic properties [9]. In the present study, we initiated VNS therapy at stage of epilepsy (development of an epileptic condition, the 4th week after pilocarpine injection) with different therapy duration (4/8 weeks) in epileptic rats. The findings indicate that VNS administered during epileptogenesis significantly reduces the frequency of SRS and shortens their duration, thereby attenuating seizure severity (Figure 1).

### 4.2 VNS Inhibits DNA Methylation

Epigenetic modifications, which influence gene activity without altering the underlying DNA sequence, play a pivotal role in epileptogenesis by inducing lasting changes in neuronal excitability [32, 33]. Among these, DNA methylation is a key regulatory mechanism that modulates gene expression through the addition of a methyl group (CH_3_) to cytosine residues, predominantly at CpG sites. This covalent modification results in the formation of 5‐methylcytosine (5‐mC), a process catalyzed by DNA methyltransferases (DNMTs) [34]. Additionally, 5‐hydroxymethylcytosine (5‐hmC), an oxidized derivative of 5‐mC, is produced by ten‐eleven translocation (TET) enzymes [35]. In the central nervous system, DNA methylation contributes to epileptogenesis by influencing synaptic plasticity and neuronal network dynamics [36]. DNA hypermethylation was confirmed in chronic epilepsy states in both animal models and epileptic tissues from patients [12, 13, 16]. Increased expression of DNMT1 and DNMT3A has been demonstrated in epileptic model and patients with temporal lobe epilepsy [13, 37, 38]. Reversed hypermethylation, attenuated seizure severity, and later onset of epileptogenesis (kindling model in mice and rats using pentylenetetrazol) associated with the application of a DNMT inhibitor 5‐Aza‐2dC [13]. In the present study, we established a model of temporal lobe epilepsy in rats characterized by SRS following pilocarpine–induced status epilepticus and initiated VNS therapy at stage of epileptogenesis (development of an epileptic condition, the 4th week after pilocarpine injection), and demonstrated VNS inhibition remarkable of the overexpression of DNMT1 and DNMT3A in epileptic rats (Figure 2). The results indicated that VNS therapy reduces the frequency of SRS and shortens the duration of seizures might via reverse the DNMT1 and DNMT3A.

Elevated global DNA methylation, such as the upregulation of 5mC in epilepsy demonstrated in a previous study [13, 37]. To determine whether alterations in DNMT1 and DNMT3A expression affected global DNA methylation and hydroxymethylation, we assessed 5‐mC and 5‐hmC levels using immunohistochemical analysis. We found significantly increased 5mC and decreased 5‐hmC immunoreactivity in the epileptic hippocampus. After 4/8 weeks of VNS therapy initiated at 4 weeks after pilocarpine‐induced status epilepticus, VNS inhibits the overexpression of 5Mc (Figure 2) and attenuates the downregulation of 5hmC (Figure 3). The results confirmed that VNS inhibited seizures at least partially via reversal of the level of DNA methylation.

### 4.3 VNS Inhibits RNA Methylation

N6‐methyladenosine (m6A) is a prevalent and reversible epigenetic modification on mRNA that provides an additional regulatory layer to gene expression [39]. The m6A methylation system involves three main components: methylation transferases (writers), demethylases (erasers), and methylated reading proteins (readers) [40]. Writers catalyze the addition of methyl groups to adenosine residues, erasers remove these modifications, and readers recognize and interpret the methylated bases to modulate RNA metabolism. Among the core methyltransferase complex, METTL3 functions as the primary catalytic enzyme, while METTL14 stabilizes METTL3 and facilitates RNA substrate recognition. Demethylases such as fat mass and obesity‐associated protein (FTO) remove m6A marks, and the loss of FTO activity results in enhanced m6A modification [40].

The role of m6A modification including methylation transferases in epilepsy is still unclear. In pentylenetetrazol (PTZ)‐induced acute seizures mice model, the level of m6A and m6A methyltransferase METTL14 is decreased by PTZ stimulation. On the other hand, in chronic posttraumatic epilepsy (PTE) model induced traumatic brain injury, increased level of m6A and downregulated of FTO was demonstrated in hippocampus of mice with posttraumatic epilepsy. The expression of METTL3 and METTL14 that regulate m6A modification were not found significant change [41]. Overexpression of FTO could alleviate epilepsy susceptibility and brain damage through mediating epigenetic up‐regulation of m6A in mice with PTE [41]. In the present study, a model of temporal lobe epilepsy in rats induced by pilocarpine was used. The upregulation of METTL3 and METTL14 in hippocampus was demonstrated in epileptic rats at 8 and 12 weeks after status epilepticus induced by pilocarpine. After 4/8 weeks of VNS therapy, VNS inhibits remarkably the overexpression of METTL3 (Figure 4) and METTL14 (Figure 5). Both enzymes are known to play critical roles in neuronal development, synaptic plasticity, and neuronal excitability [42]. Recent studies have implicated dysregulated m6A methylation in abnormal neural circuit activity and seizure susceptibility [40]. Our findings that VNS modulates the expression of METTL3 and METTL14 suggest that m6A methylation may contribute to the antiepileptic effects of VNS, potentially by reshaping transcriptomic profiles associated with hyperexcitability. Together, these results suggested that VNS therapy inhibited epileptic seizures might be involved with reversal of methyltransferase METTL3 and METTL14.

## 5 Conclusion

Our data suggest that VNS therapy initiated during epileptogenesis can decrease the frequency and severity of SRS in epileptic rats. Specifically, we demonstrate that VNS therapy decrease of 5‐mC, DNMT1, DNMT3A, METTL3, and METTL14, as well as increase of 5‐hmC in epileptic rats. DNA/RNA methylation through the DNMT1/3A and METTL3/METTL14 pathway might be therefore a potential target of VNS therapy for the treatment of epilepsy. Given the emerging role of methylation dynamics in neuronal excitability and seizure susceptibility, targeting these pathways may offer novel translational opportunities. Future studies are warranted to explore the therapeutic potential of combining VNS with epigenetic interventions in human epilepsy.

## Author Contributions

All authors contributed to the study conception and design. Zhonghua Xiong and Tianfu Li conceived and designed the experiments, authored, and reviewed drafts of the paper. Zhonghua Xiong wrote the original draft. Zhonghua Xiong and Jing Zhang analyzed the data. Tianfu Li reviewed and edited the final draft. Zhonghua Xiong, Jing Zhang, Qinqin Deng, Minghui Wang performed the experiments and prepared Figures and/or tables. Tianfu Li approved the final draft. All authors contributed to the article and approved the submitted version.

## Ethics Statement

This study was performed in line with the principles of the Declaration of Helsinki and complied with the ARRIVE guidelines. Approval for the study was obtained from the Animal Experiments and Experimental Animal Welfare Committee of Capital Medical University (AEEI‐2021‐005).

## Consent

The authors have nothing to report.

## Conflicts of Interest

The authors declare no conflicts of interest.

## Supporting information


Data S1.

## Data Availability

The datasets generated during and/or analyzed during the current study are available from the corresponding author on reasonable request.
