# Supplementary material for: Vagus Nerve Stimulation Inhibits DNA and RNA Methylation in a Rat Model of Pilocarpine‐Induced Temporal Lobe Epilepsy
Source: CNS Neurosci Ther. 2025 Jun 18;31(6):e70484. doi: 10.1111/cns.70484 (PMC12177201; doi:10.1111/cns.70484)

Supplementary material. Uncropped Gels and Blots images.

Full unedited gel/blot for Figure 2K

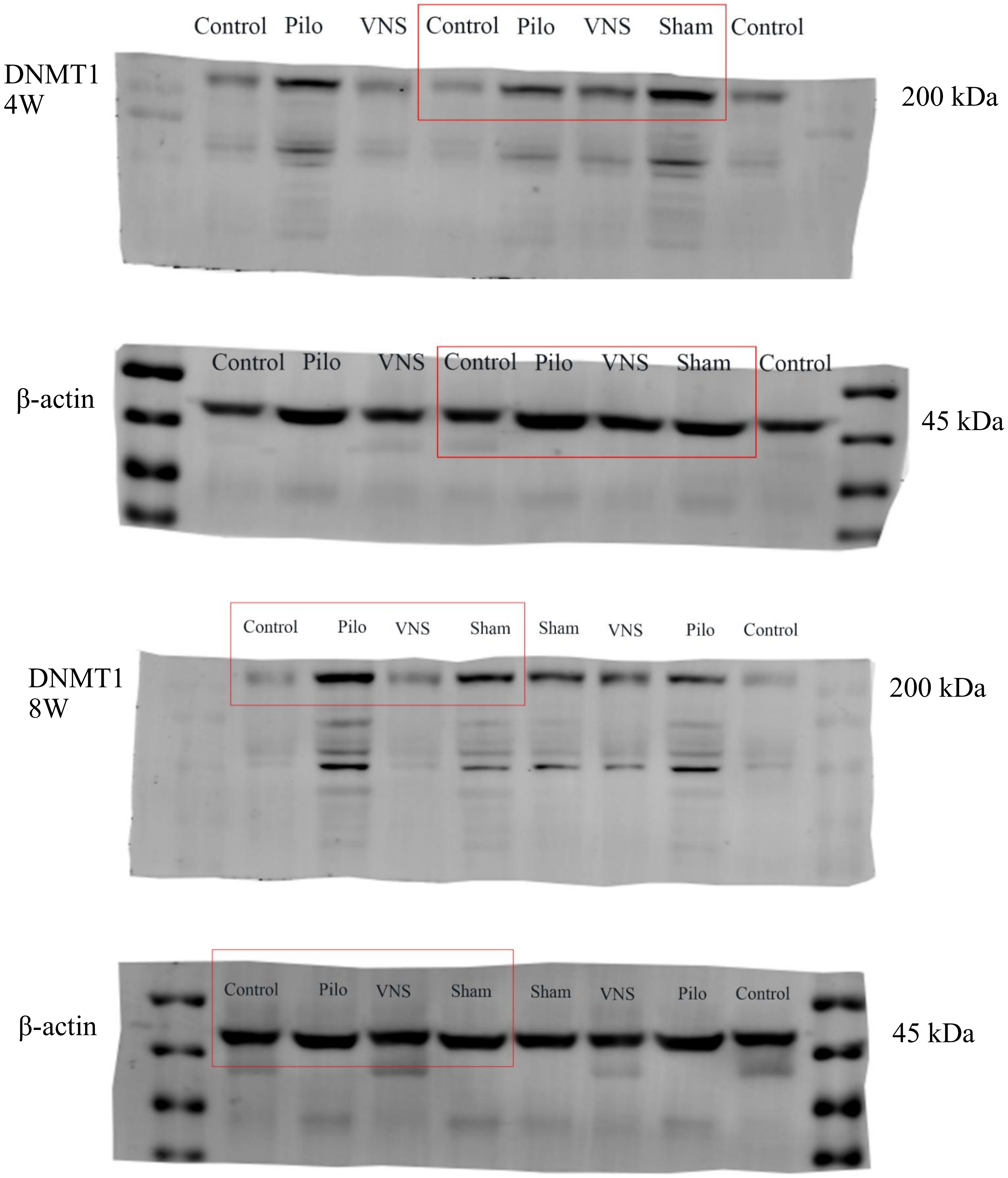

Full unedited gel/blot for Figure 2K

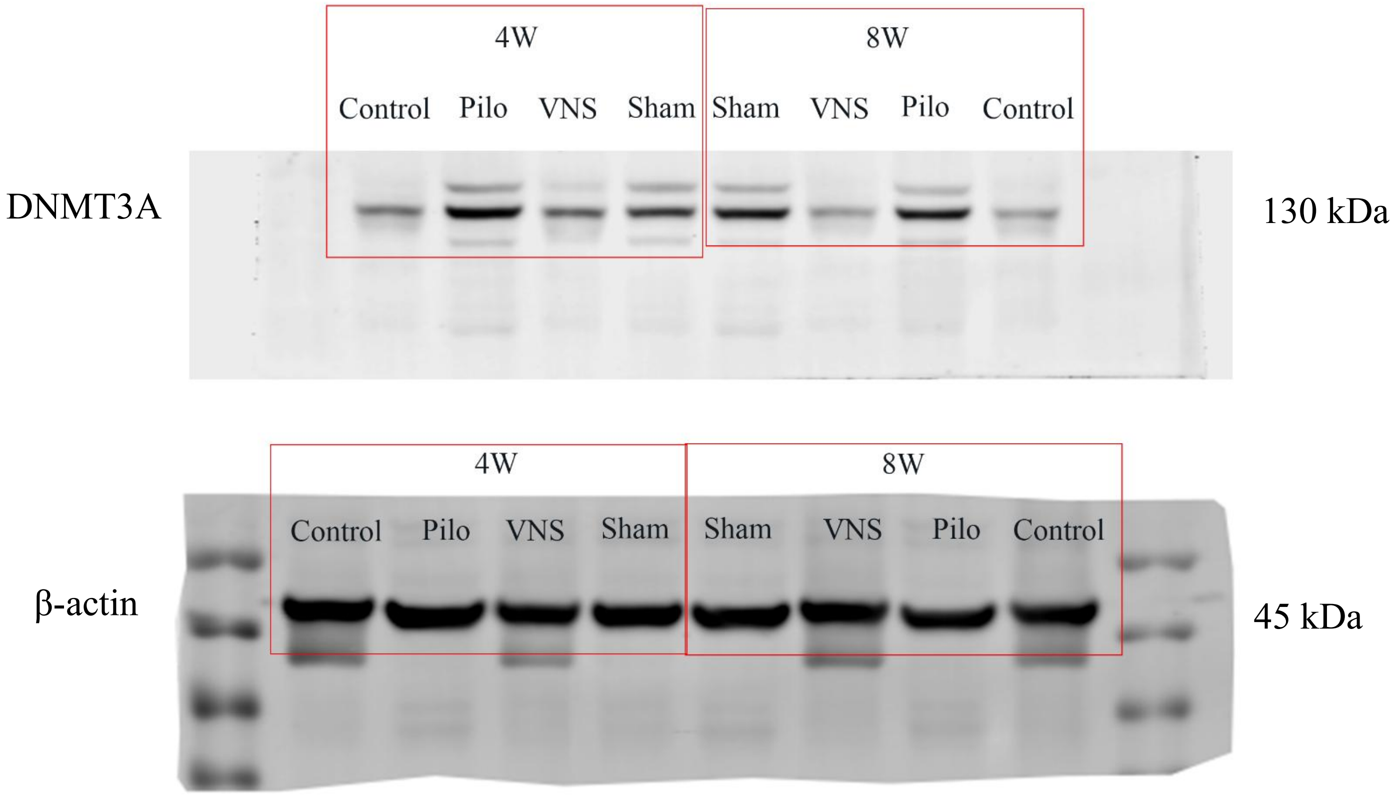

Full unedited gel/blot for Figure 4K

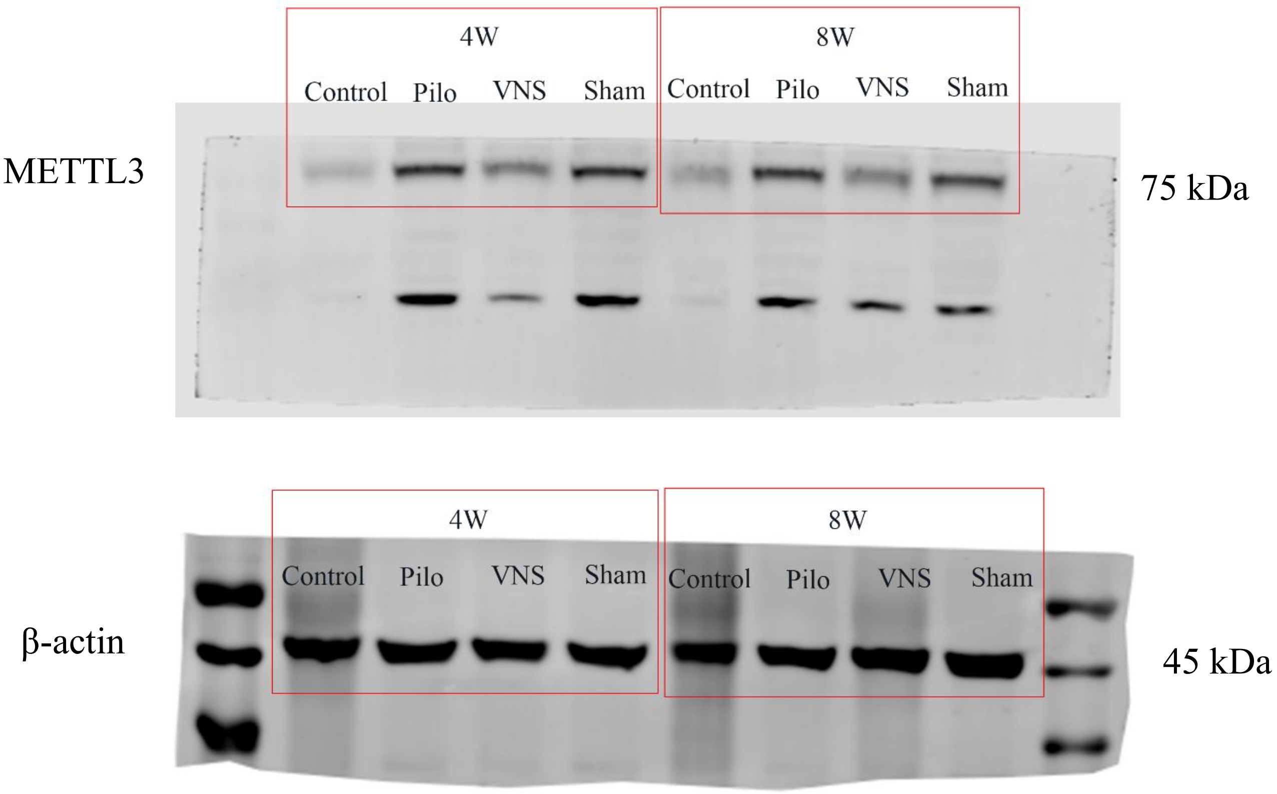

Full unedited gel/blot for Figure 5K

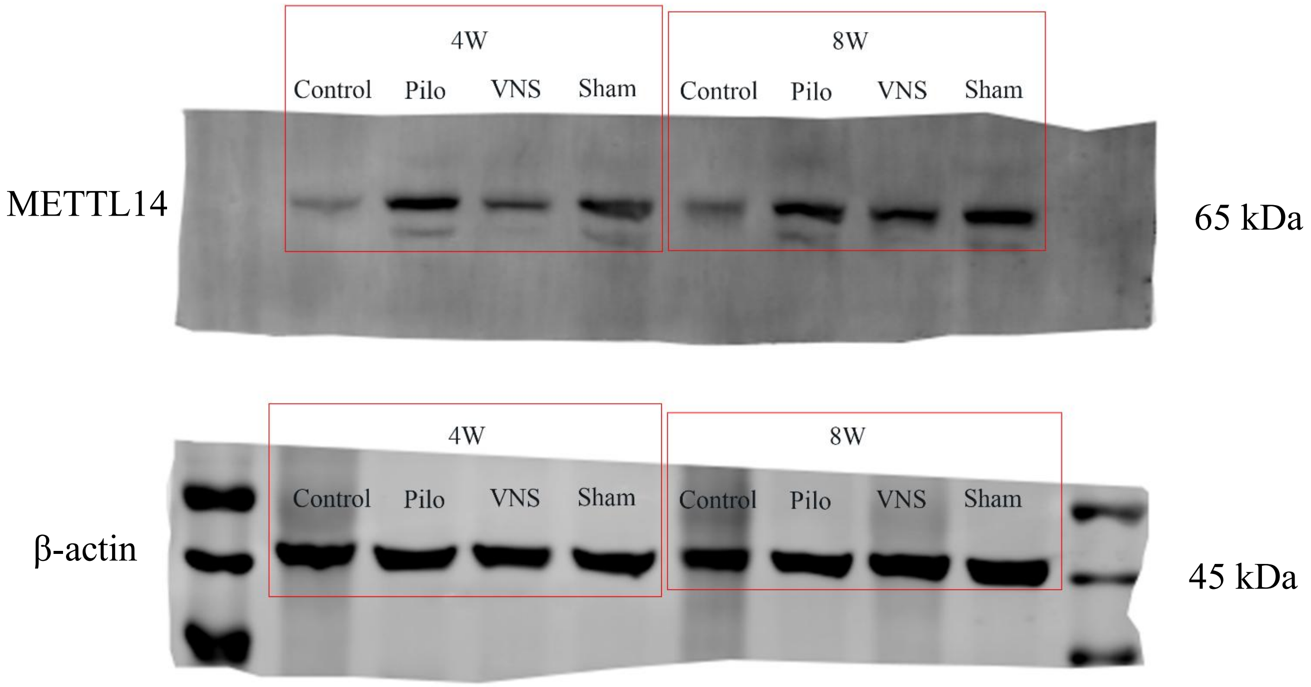

Supplement: Supplementary file 1 — Data S1. [file CNS-31-e70484-s001.pdf]
